# Enhanced Strength of Al-10Ce-3Mg-5Zn Heat-Resistant Alloy by Combining Extrusion and Heat Treatment

**DOI:** 10.3390/ma18081706

**Published:** 2025-04-09

**Authors:** Haiyang Zhang, Zeyu Li, Daihong Xiao, Mingdong Wu, Yang Huang, Wensheng Liu

**Affiliations:** National Key Laboratory of Science and Technology on High-Strength Structural Materials, Central South University, Changsha 410083, China; 223311047@csu.edu.cn (H.Z.); 16620176815@163.com (Z.L.); mmingshiddong@csu.edu.cn (M.W.); youngh@csu.edu.cn (Y.H.); liuwensheng@csu.edu.cn (W.L.)

**Keywords:** Al-Ce-Mg-Zn alloy, Al_11_Ce_3_ phase, bimodal microstructure, twin, mechanical properties

## Abstract

The existing Al-Ce heat-resistant alloys are not extensively utilized in high-temperature applications due to their poor room-temperature mechanical properties. In this study, the Al-10Ce-3Mg-5Zn alloy was enhanced using hot extrusion and heat treatment. The as-extruded alloy exhibited bimodal intermetallic compounds and grain structures. Additionally, high-density microcracks and twins were observed in the micron-sized intermetallic compounds. Compared with the as-cast state, the as-extruded alloy demonstrated a higher ultimate tensile strength (UTS) of 317 MPa and better elongation of 11.0%. Numerous nano-sized T phases precipitated in the α-Al matrix after the heat treatment, contributing to a further rise in UTS (365 MPa). The high strength of the alloy is primarily due to its strong strain hardening capacity, fine grain strengthening, and precipitation strengthening effect. The change in elongation mainly results from the expansion of pre-existing microcracks, twin deformation, and microstructural refinement. The heat-treated alloys exhibited superior strength retention ratios at elevated temperatures (64% at 200 °C) compared to conventional heat-resistant aluminum alloys. The results of this paper indicate that hot extrusion and heat treatment are effective for developing heat-resistant Al-Ce alloys with high room-temperature strength, offering a simple process suitable for industrial production.

## 1. Introduction

The growing development of new energy vehicles, rail transit, and the aerospace industry has driven a rapid increase in the demand for aluminum alloys [1,2,3]. However, the mechanical properties of aluminum alloys decline sharply during service at 200–300 °C [4,5,6], which is the main reason why they are challenging to use in high-temperature fields. Recently, Al-Ce alloys designed around the eutectic reaction of Al and Ce at 642 °C have recently gained attention for their excellent thermodynamic stability and mechanical properties at elevated temperatures [7,8,9,10]. The solubility of Ce in Al is less than 0.005 wt.% when near the eutectic temperature; this leads to the formation of a thermodynamically stable Al_11_Ce_3_ intermetallic compound during solidification [11,12]. Consequently, binary eutectic and near-eutectic Al-Ce alloys exhibit superior castability and thermal stability. However, the mechanical properties of conventionally cast Al-Ce alloys at room temperature are not excellent [13,14]. Weiss et al. [15] studied the mechanical properties of binary Al-Ce alloys at room temperature. Their results showed that alloy strength increased when the Ce content increased, and the maximum ultimate tensile strength (UTS) was only 163 MPa. The relatively low room-temperature strength limits the wide application of Al-Ce alloys as heat-resistant aluminum alloys.

In general, alloying is a simple way to improve the mechanical properties of materials [16,17]. Elements such as Cu, Mg, and Si are incorporated into Al-Ce alloys to enhance properties through solid solution strengthening and precipitation strengthening [18,19]. For instance, Wang et al. [19] incorporated 2 wt.% Cu and 0.8 wt.% Mg into Al-12Ce, resulting in a 16% increase in the UTS of the alloy, from 163 MPa to 179 MPa. Czerwinski et al. [20] added 3 wt.% Si to Al-5Ce-0.5Mg alloy and the UTS of the alloy significantly increased from 113.5 MPa to 142 MPa. Moreover, the addition of Y and Zr elements in Al-Ce alloy can improve the morphology of the Al_11_Ce_3_ phase, thereby improving the strength of the alloy [17,21]. Our previous study has found that the addition of 5 wt.% Zn in Al-10Ce-3Mg alloy can enhance the second phase strengthening of the alloy and improve the properties of the alloy [22]. Unfortunately, the addition of a high ratio of strengthening elements is not conducive to the ductility of Al-Ce alloys. For example, the addition of 10 wt.% Mg to Al-8Ce results in the formation of a large primary Al_11_Ce_3_ intermetallic phase, which in turn leads to stress concentration and a subsequent decrease in the elongation from 19% to 1% [23].

Grain refinement and intermetallic compound refinement have been proven to effectively improve the plasticity of Al-Ce alloys [24,25]. Ultra-fine microstructures can be obtained directly through various advanced techniques, including permanent magnet stirring [26,27], Sonoprocessing [28], spray forming [29], and laser additive manufacturing [30,31,32]. Wang et al. [27] employed permanent magnet stirring technology to transform the eutectic structure of the Al-5Ce alloy from a flaky to a fibrous morphology, enhancing its UTS from 90 MPa to 136 MPa. Zhou et al. [31] produced the Al-10Ce alloy with a highly refined eutectic Al_11_Ce_3_ structure by means of additive manufacturing technology. The room-temperature strength of this alloy is 319 MPa, which is much higher than that of the cast alloy (152 MPa). However, these experimental techniques are complicated and expensive. The hot extrusion process is an effective method for refining the microstructure of an alloy and eliminating certain casting defects [33,34]. In comparison to the above methods, the hot extrusion process is more cost-effective. Zhang et al. [35] studied the effect of extrusion on the microstructure and properties of the Al-9Ce alloy. The results showed that the thermal stability of the Al-Ce alloy was independent of its preparation methods (extrusion and casting) and that its tensile strength increased by 62% after extrusion. Therefore, hot extrusion can effectively enhance the properties of Al-Ce alloys by refining grains and optimizing the distribution of strengthening phases.

Although the superior mechanical properties of extruded Al-Ce-based alloys have garnered considerable attention, the majority of research has primarily concentrated on binary Al-Ce alloys. The microstructure and mechanical properties of Al-Ce alloys containing alloying elements such as Mg and Zn under hot deformation conditions remain unexplored. Furthermore, heat treatment techniques such as solution treatment and aging improve the solubility of alloying elements and facilitate the precipitation of nano-scale strengthening phases, significantly influencing the microstructure and properties of conventional aluminum alloys [36,37,38]. Chu et al. [39] carried out a single-stage solid solution aging treatment on the Al-Zn-Mg-Cu alloy, in which the η nano-strengthening phases were precipitated in the crystal, which enhanced the alloy’s mechanical properties. Consequently, the combination of hot extrusion and an appropriate heat treatment may be more conducive to achieving a uniform structure and high mechanical properties in Al-Ce alloys.

Based on the above research, this paper investigates the influence of hot extrusion and heat treatment on the microstructure, room-temperature mechanical properties, and high-temperature mechanical properties of the Al-10Ce-3Mg-5Zn alloy. In addition, the strengthening mechanism of the as-extruded Al-10Ce-3Mg-5Zn alloy following heat treatment is discussed, which provides a foundation for the development of a novel high-strength and heat-resistant aluminum alloy. Moreover, it also provides a feasible idea for breaking through the bottleneck of the high-temperature performance of aluminum alloy and realizing the efficient utilization of rare earth cerium resources.

## 2. Materials and Methods

The alloy with a design composition of Al-10Ce-3Mg-5Zn-0.1Zr-0.1Y was subjected to complete melting in a resistance furnace at 800 °C utilizing pure Al (99.99 wt.%), Mg (99.99 wt.%), Zn (99.99 wt.%), along with Al-30 wt.% Ce, Al-5 wt.% Zr, and Al-10 wt.% Y intermediate alloys as raw materials [22]. The aluminum liquid was fully stirred to achieve a uniform distribution of elements, and then argon was introduced for refining. After maintaining at 780 °C for 20 min, the impurities and oxide scale in the melt were removed, and the molten aluminum was uniformly poured into a 120 mm diameter copper mold. The actual chemical composition of the ingot was determined through inductively coupled plasma atomic emission spectrometry (ICP-AES, Spectro Blue SOP, SPECTRO, Kleve, Germany) and was found to be Al-10.5Ce-2.9Mg-4.9Zn-0.1Zr-0.1Y (wt.%). The as-cast alloy was homogenized at 470 °C for 12 h and then machined into a cylindrical extruded billet with a diameter of 98 mm and a height of 80 mm. The billet was subjected to hot extrusion at 450 °C with an extrusion ratio of 13:1. Following the hot extrusion process, the final formed 48mm (width) × 12mm (thickness) sheet was water-cooled to room temperature.

The phases of the alloy were detected using X-ray diffraction (XRD, D8 Advance, Bruker, Bremen, Germany) with Cu Kα radiation. The scanning range was configured from 10° to 90° with a step size of 5°/min. In order to observe the microstructure of the alloy, its surface was ground with SiC sanders to a 2000 mesh and then polished with Al_2_O_3_ polishing powder. The microstructure of the sample was characterized by means of scanning electron microscopy (SEM, MIRA4 LMH, TESCAN, Brno, Czech Republic). The size of the intermetallic phase in the alloy was calibrated using the Image Pro Plus 6.0 software. An electron probe microanalyzer (EPMA, JXA-8230, JEOL, Tokyo, Japan) was used to analyze the distribution of elements in the alloy. The mechanically polished sample was then electropolished to prepare the electron backscatter diffraction sample. The electron backscattered diffraction (EBSD) observations were performed using an SEM (Regulus 8230, Hitachi, Tokyo, Japan) equipped with a backscattered electron detector (Oxford Instrument, Oxford, UK). The EBSD data were subjected to analysis using the AZtec 5.1 software. In order to facilitate transmission electron microscopy characterization, a sample with a diameter of 3 mm was mechanically thinned to a thickness of approximately 100 μm, and then the specimen was subjected to electrolytic double-jet thinning (Gatan 695, Gatan, Pleasanton, CA, USA). A transmission electron microscope (TEM, Talos F200X, Thermo Fisher Scientific, Waltham, MA, USA) was used to capture the TEM images and selected area electron diffraction (SAED) images at 200 kV.

To evaluate the effect of T6 heat treatment on the mechanical properties of the as-extruded alloy, the alloy underwent solution treatment at 515 °C for 1 h, followed by water quenching and aging at 150 °C. The peak aging condition was identified by measuring the Vickers hardness using a Vickers hardness tester (200HV-5, Huayin, China) under a load of 5 kg and a duration of 15 s. The final value was determined by averaging five points. The samples were machined into flaky tensile specimens with dimensions of 80 mm in length, 6 mm in width, and 2 mm in thickness. The gauge length was parallel to the extrusion direction for the deformed alloy. The ambient mechanical property tests were performed at room temperature using a mechanical testing machine (Instron 3609, Instron, Norwood, MA, USA) at a strain rate of 2 mm/min according to GB/T228.1-2010 [40]. The samples were tested three times in each state. To evaluate the heat resistance of the alloy, high-temperature tensile tests were conducted on the dog-bone-shaped specimens following deformation and heat treatment. The specimens were designed with dimensions of 32 mm in length, 3.5 mm in width, and 1.5 mm in thickness. All the tests were carried out at 200 °C, 260 °C, and 300 °C using a Zwick/Roell Z100 testing machine (Zwick Roell, Ulm, Germany) at a strain rate of 1 mm/min. To investigate the influence of the microstructure on the fracture behavior of the alloy, the microstructure near the fracture surface was examined using an SEM.

## 3. Results

### 3.1. The Microstructures of the Al-10Ce-3Mg-5Zn Alloy

**Figure 1:**
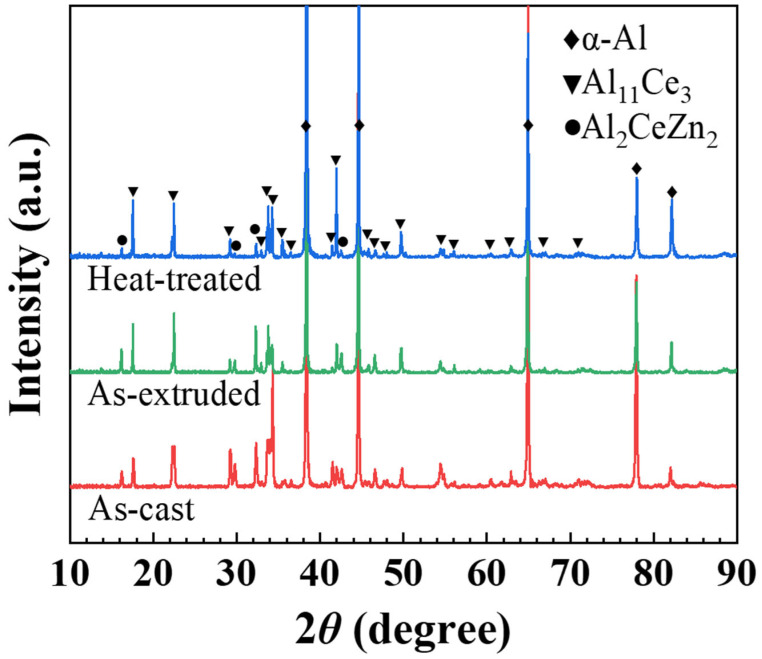
The XRD diffraction patterns of the Al-10Ce-3Mg-5Zn alloy.

Figure 1 illustrates the typical XRD patterns of the alloy in the as-cast, as-extruded, and heat-treated states. The majority of peaks in all the alloys were attributed to the α-Al matrix and Al_11_Ce_3_ intermetallic compound. However, a small number of peaks of Al_2_CeZn_2_ were also detected. The analysis reveals that the alloy mainly consists of an α-Al phase, an Al_11_Ce_3_ phase, and an Al_2_CeZn_2_ phase. It can be concluded that the phase types in the alloy remain largely unaffected by the hot extrusion and heat treatment processes.

**Figure 2:**
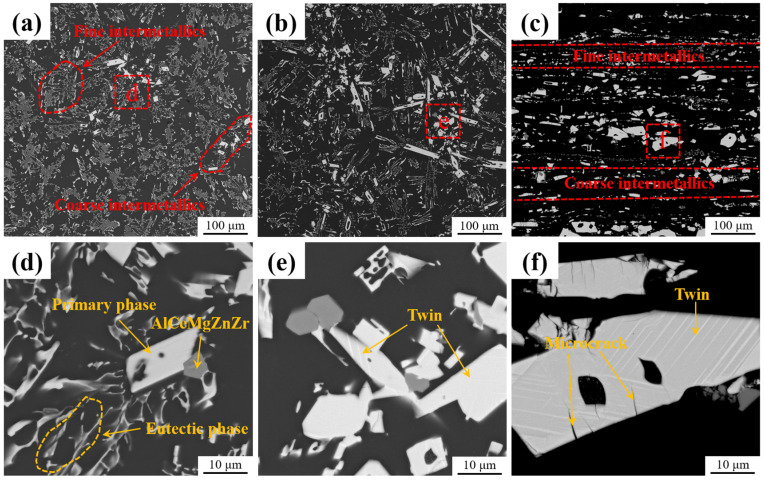
BSE-SEM images of the (**a**,**d**) as-cast, (**b**,**e**) as-homogenized, and (**c**,**f**) as-extruded microstructures of the Al-10Ce-3Mg-5Zn alloy.

Figure 2 shows the backscattered electron (BSE)-SEM micrographs of the alloys in the as-cast, as-homogenized, and as-extruded conditions. It can be observed that the Al_11_Ce_3_ intermetallic compound in the microstructure of the alloy e is characterized by a bright contrast, while the α-Al matrix is distinguished by a dark contrast (Figure 2a). The fine-layered Al_11_Ce_3_ intermetallic compound is uniformly dispersed in the α-Al matrix. The coarse Al_11_Ce_3_ intermetallic compounds are irregular blocks (Figure 2d), which are randomly dispersed within the α-Al matrix. Figure 2b is an image of the as-cast alloy after homogenization at 470 °C for 12 h. The microstructure of the alloy exhibits no significant changes, which can be attributed to the excellent thermal stability of the Al_11_Ce_3_ phase. However, further research on the large-scale intermetallic phase found that its surface is decorated with white stripes (Figure 2e). Subsequent research determined that the white stripes are twins. After hot extrusion, the intermetallic compound rearranges along the extrusion direction. The microstructure exhibits a typical lamellar structure and is alternately arranged in two different sizes of intermetallic compound (Figure 2c). Observing the coarse intermetallic compound at a higher magnification showed that the bulk phase is partially fragmented and exhibits a considerable number of microcracks and twins on the surface, as shown in Figure 2f. Moreover, trace amounts of Mg-, Zn-, and Zr-rich intermetallic compounds were found in the vicinity of the Al_11_Ce_3_ phases. This is attributed to the solidification behavior of Al_11_Ce_3_ phases, which causes the accumulation of Mg, Zn, and Zr at the solid–liquid interface [41].

In general, during hot extrusion, the α-Al matrix undergoes plastic flow along the extrusion direction. Due to its brittle nature, Al_11_Ce_3_ experiences stress during the extrusion process. This leads to the complete fragmentation of the small-scale phase, incomplete fragmentation of the coarse intermetallic compound, and formation of high-density microcracks on their surfaces. Meanwhile, the fragmented Al_11_Ce_3_ phase rotates in response to the flow of the α-Al matrix, ultimately aligning with the extrusion direction.

**Figure 3:**
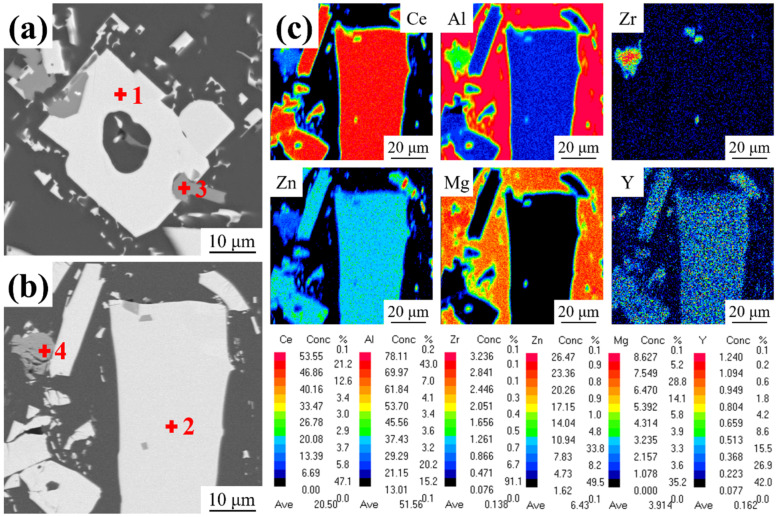
EPMA elemental maps of the Al-10Ce-3Mg-5Zn alloy: (**a**) scanning morphology of the as-cast alloy, (**b**) scanning morphology of the as-extruded alloy, and (**c**) elemental mapping of (**b**). Points 1–4 are used for the EPMA analysis.

Figure 3 shows the EPMA results in the as-cast and as-extruded alloys. The chemical composition of the points depicted in Figure 3a,b is shown in Table 1. The results of our point analysis of the two phases in the as-cast alloy indicate that the white phase is an Al_11_Ce_3_ phase containing Zn, while the gray phase is an AlCeMgZnZr phase (Figure 3a). Comparing the point analysis results of the phases in both the as-cast and as-extruded alloys, it can be found that the element distribution of the same phase basically remains unchanged. It has been demonstrated that hot extrusion does not alter the type of phase that is present in an alloy, but it does affect the morphology of the phase. Furthermore, it has been shown that intermetallic compound exhibits good thermal stability. The surface scanning results of the as-extruded alloy are shown in Figure 3c. The Al_11_Ce_3_ phase predominantly contains the Ce element, while the Mg element is mainly dissolved in the Al matrix, contributing to the solid solution strengthening effect. The Zn element is mainly concentrated in the Al_11_Ce_3_ phase, with some of the Al_11_Ce_3_ reacting with Zn to form the Al_2_CeZn_2_ phase. This observation aligns with the findings of previous studies [22].

**Figure 4:**
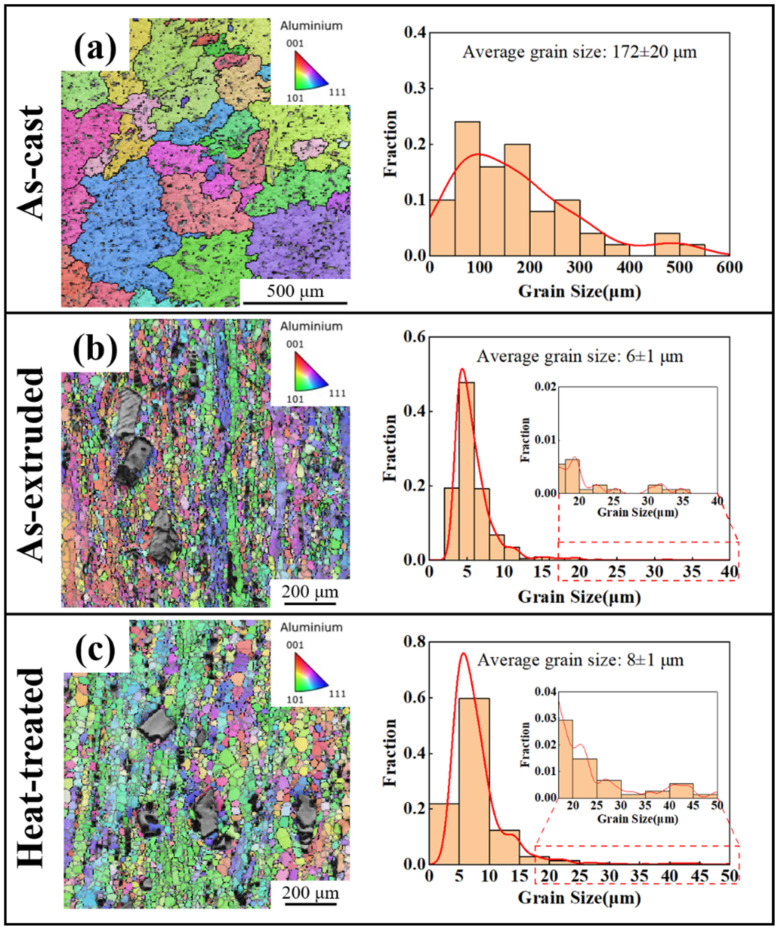
EBSD images and average grain size distributions of the (**a**) as-cast, (**b**) as-extruded, and (**c**) heat-treated alloys.

**Figure 5:**
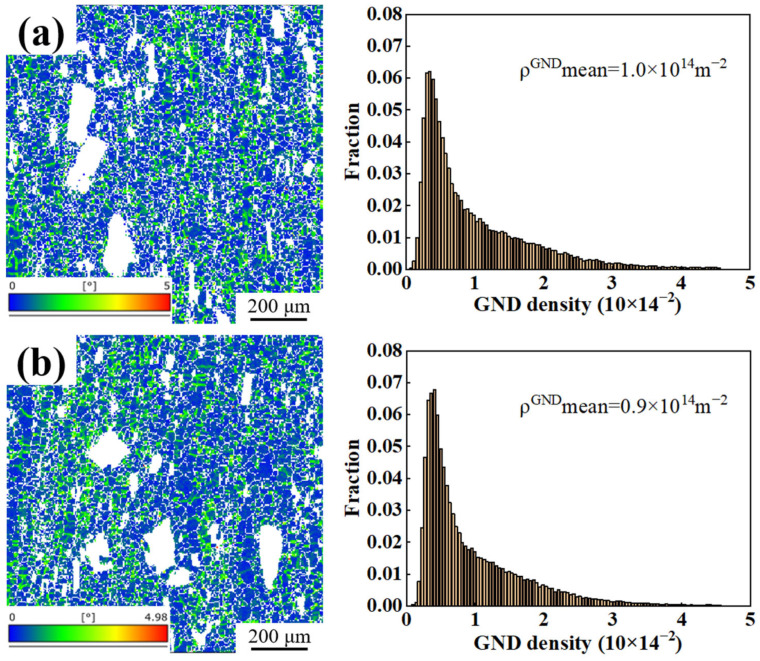
KAM maps and GND distributions of the (**a**) as-extruded and (**b**) heat-treated alloys.

**Figure 6:**
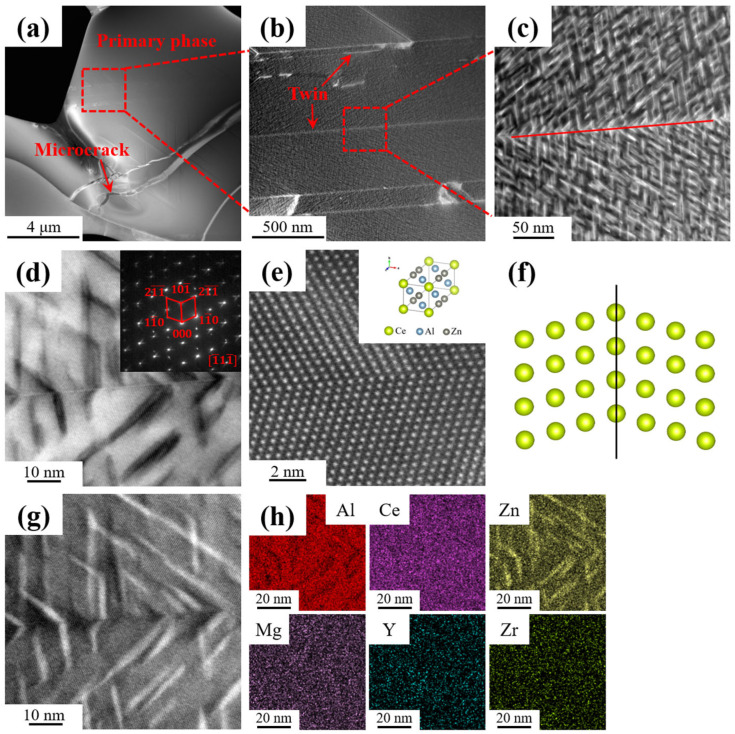
TEM investigations of the as-extruded alloy: (**a**) DF micrograph of the Al_11_Ce_3_ phase; (**b**) DF image of the area marked by the square in (**a**); (**c**) DF image of the area marked by the square in (**b**); (**d**) bright-field (BF) image and corresponding SAED patterns of the primary phase; (**e**) HRTEM image and atomic arrangement model of the Al_2_CeZn_2_ phase; (**f**) reflection symmetry between neighboring components of twins corresponding to (**e**); (**g**) high-angle annular dark-field (HAADF)-STEM image of twins; and (**h**) corresponding EDS element maps of (**g**).

Figure 4 presents the EBSD images along with the average grain size distributions of the as-cast, as-extruded, and heat-treated alloys. The unidentified regions in the figure are intermetallic compounds. It can be seen that all the alloys exhibit an equiaxed structure and do not show an obvious preferred grain orientation. Following hot extrusion, the grain size of the alloy was significantly reduced from 172 ± 20 μm (Figure 4a) to 6 ± 1 μm (Figure 4b). This indicates that obvious recrystallization occurred in the alloy throughout the hot extrusion process. After heat treatment, the extruded α-Al recovered further and recrystallized, resulting in grain growth. As shown in Figure 4c, the average grain size of the heat-treated alloy was measured to be 8 ± 1 μm. Moreover, the grain size distribution of the as-extruded and heat-treated alloys is roughly bimodal. The grains surrounding the coarse intermetallic compound are several microns, while those surrounding the fine intermetallic compound are tens of microns.

Figure 5 illustrates the kernel average misorientation (KAM) maps and the distributions of geometrically necessary dislocations (GNDs) for both the as-extruded and heat-treated alloys. The white areas in the figure are the intermetallic phases. It can be observed that after hot extrusion, a deformation zone with a high dislocation density and significant orientation gradient forms near the intermetallic particles (Figure 5a). Furthermore, the degree of plastic deformation is higher near coarse intermetallic compounds. The stress concentration within the deformation zone generates the essential driving force for recrystallization. The heat-treated alloy exhibits a lower GND density than that of the as-extruded alloy (Figure 5b). The reason for this is that the alloy continues to recover and recrystallize during the heat treatment, resulting in the release of deformation storage energy and a decrease in GND density.

The intermetallic compound in the as-extruded alloy was examined in greater detail using TEM. The TEM dark-field (DF) image of the large-scale intermetallic phase in the alloy is shown in Figure 6a. It can be clearly seen that the phase was partially broken and there were some microcracks on the surface. At the same time, some narrow stripes emanating from cracks were observed in the middle of the phase (Figure 6b). By observing the phase with high multiples, it was found that a considerable number of nano-scale needle-like phases were regularly arranged. The EDS results of Figure 6g are shown in Figure 6h, which reveals that the acicular phase is enriched in Zn. This result demonstrated that Zn replaced a portion of the Al, forming the Al_2_CeZn_2_ phase within the Al_11_Ce_3_ phase. Interestingly, the direction of the distribution of the needle-like phase changed on both sides of the narrow stripe, as shown in Figure 6c. To gather additional information, diffraction spots for the corresponding phase were obtained by placing the direction of the electron beam incidence parallel to the axis $\left[\bar{1}1\bar{1}\right]$ (Figure 6d). It could be clearly seen that the two sets of the same diffraction spots were symmetrically distributed. Figure 6e presents the high-resolution transmission electron microscopy (HRTEM) image near the stripe. The arrangement of Ce atoms is one of mirror symmetry, which proves that the stripe is a twin boundary. For further analysis, the particles in Figure 6e were abstracted as small circles, as shown in Figure 6f. The image clearly demonstrates the presence of twin planes.

**Figure 7:**
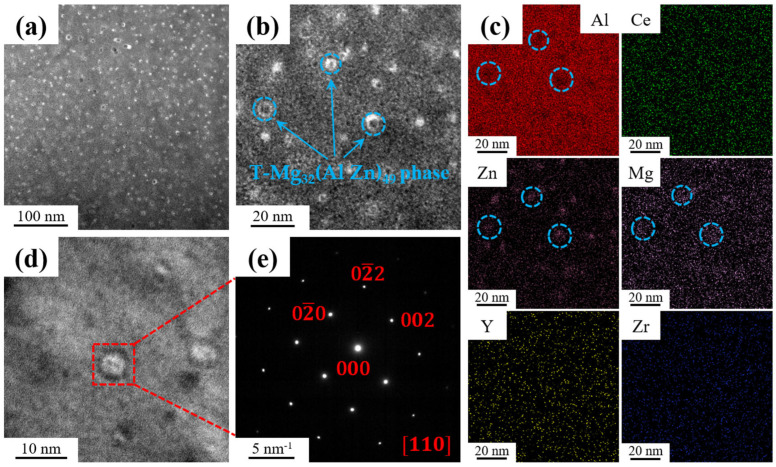
TEM images of the heat-treated alloy: (**a**,**b**) HAADF-STEM image of T phase; (**c**) corresponding EDS element maps of (**b**); (**d**) HRTEM image of T phase; and (**e**) corresponding SAED patterns of (**d**).

The microstructure of the heat-treated samples was characterized using HAADF-STEM and EDS. Figure 7a displays the HAADF-STEM image of the sample, where a significant number of fine, spherical precipitates are evenly distributed within the α-Al matrix. These precipitates have an average diameter of several nanometers, as shown in Figure 7b. Figure 7c shows the distribution of the elements in Figure 7b. The areas marked by blue circles indicate Mg and Zn enrichment at the spherical precipitates. The HRTEM image of the spherical precipitation and the SAED pattern along the [110] direction are shown in Figure 7d,e, respectively. They indicate that the spherical precipitate in the α-Al matrix is a T-Mg_32_(Al Zn)_49_ phase.

### 3.2. Mechanical Properties and Fracture Morphologies

**Figure 8:**
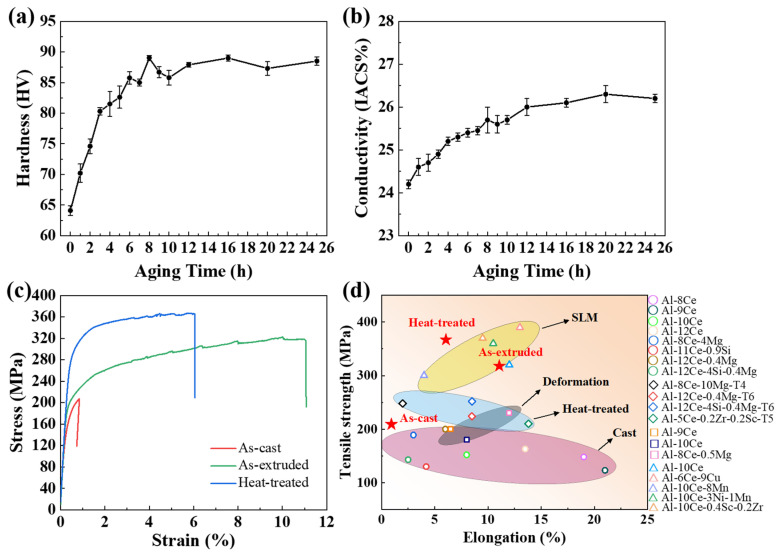
(**a**) Age hardening curve of the as-extruded alloy; (**b**) effect of aging time on the conductivity of the as-extruded alloy; (**c**) engineering tensile stress–strain curves of the as-cast, as-extruded, and heat-treated alloys at 25 °C; (**d**) comparison of the UTS and EL between the present alloys and other Al-Ce alloys produced using various methods [7,23,31,42,43,44].

Figure 8a,b display the changes in the Vickers hardness and conductivity of the as-extruded alloy during aging at 150 °C. It is evident that the alloy exhibits the phenomenon of aging hardening. The hardness and conductivity of the alloy showed a tendency to rise abruptly and then slowly as the aging time increased. The hardness of the alloy reached a peak of 89 HV at 8 h, after which it decreased at a gradual rate. Its conductivity value is in the range of 24–27% IACS. Figure 8c shows the tensile stress–strain curves at 25 °C for the as-cast, as-extruded, and heat-treated alloys. The ultimate tensile strength (UTS) and yield strength (YS) of the as-cast alloy are 212 ± 5 MPa and 172 ± 2 MPa, respectively, but the elongation (EL) of the alloy is only 0.9 ± 0.1%. After hot extrusion, the UTS and YS of the alloy were enhanced to 317 ± 4 MPa and 205 ± 1 MPa, respectively, and the EL improved significantly to 11.1 ± 0.4%. The UTS and YS of the heat-treated alloys increased by 15.8% and 42.2% compared with the as-extruded alloy, reaching 367 ± 5 MPa and 292 ± 2 MPa, respectively. However, the EL decreased to 6.0 ± 0.3%. Moreover, the heat-treated alloy had the same strength at room temperature as conventional heat-resistant aluminum alloys such as AA2618 and AA4032 (approximately 380 MPa) [23].

Figure 8d presents the comparative analysis of the mechanical properties of the studied alloys at room temperature with those of Al-Ce alloys obtained through deformation, selective laser melting (SLM), casting, and heat treatment processes [7,23,31,42,43,44]. As can be seen from the pink area in Figure 8d, as-cast Al-10Ce-3Mg-5Zn alloy has higher tensile strength but lower elongation compared to other cast Al-Ce alloys. The comprehensive properties of the as-extruded Al-10Ce-3Mg-5Zn alloy are higher than those of the deformed and heat-treated Al-Ce alloys but lower than those of most of the alloys produced by means of SLM. The heat-treated alloy has the same strength at room temperature as the alloys produced by means of SLM. Compared with the as-cast Al-10Ce-3Mg-5Zn alloy, the elongation is also greatly improved. Furthermore, the SLM process is associated with a number of disadvantages, including a high cost, complexity, and difficulty in mass production. Consequently, it is challenging to use it widely in industrial production. The Al-10Ce-3Mg-5Zn alloy, produced through hot extrusion and heat treatment, offers a practical method for fabricating Al-Ce alloys with high mechanical properties.

**Figure 9:**
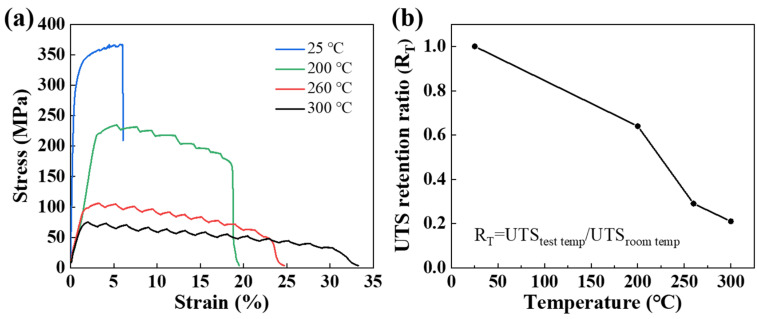
(**a**) Engineering stress–strain plots of the heat-treated Al-10Ce-3Mg-5Zn at high temperatures; (**b**) UTS retention ratio of the heat-treated Al-10Ce-3Mg-5Zn, expressed as a ratio of UTS measured at the test temperature to UTS measured at room temperature.

In order to evaluate the high-temperature mechanical properties of the heat-treated alloy, tensile tests were conducted at various temperatures (200, 260, and 300 °C). It was demonstrated that the alloy’s mechanical properties undergo a pronounced decline at elevated temperatures (Figure 9a). The UTS at room temperature (367 MPa) was observed to decrease to 234 MPa, 106 MPa, and 77 MPa at 200, 260, and 300 °C, respectively. In addition, the UTS retention rate of the heat-treated alloy at room temperature was calculated to be 64% at 200 °C, 29% at 260 °C, and 21% at 300 °C (Figure 9b). It has been demonstrated that the reduction in alloy strength with increasing temperatures is primarily attributable to the coarsening of the strengthening phase, the acceleration of atomic diffusion, and an increased concentration of vacancies at elevated temperatures [35]. Zhao et al. [5] have shown that when the temperature exceeds 200 °C, the T phases undergo coarsening. Therefore, the coarsening of a significant number of T phases in the heat-treated Al-10Ce-3Mg-5Zn alloy at elevated temperatures is the primary factor responsible for the decline in its high-temperature performance. However, compared with the traditional heat-resistant aluminum alloys AA4032 (whose UTS retention rate is 24% at 200 °C), AA2618 (whose UTS retention rate is 23% at 260 °C), and AA7075 (whose UTS retention rate is 15% at 300 °C) [45], the heat-treated Al-10Ce-3Mg-5Zn alloy still has a good UTS retention ratio. Moreover, considering the high room-temperature strength of the studied alloy, it has a strong potential for application in a high-temperature environment.

Interestingly, the stress–strain curves of the alloys at room temperature exhibited serrations (Figure 8c). A distinct serration behavior was also observed during tensile testing at medium to high temperatures (Figure 9a). Previous studies have indicated that serrated flow behavior is observed in many metal materials [46]. In general, this mechanism can be attributed to two main reasons: (i) deformation twinning and (ii) the interaction between the solute atoms and movable dislocations [47,48]. At room temperature, deformation twinning is considered to be the primary cause of serrated flow [49]. In this study, twins appeared in the intermetallic compound of the as-cast alloy after homogenization (Figure 2e). Therefore, it can be logically inferred that the formation of twins leads to serrated stress–strain curves at room temperature. Throughout the deformation process, twins can reduce the concentration of stress. The repetitive twinning of the alloy and the accumulation of tensile stress result in the formation of serrations until the tensile fracture of the sample occurs. Furthermore, the serrated flow behavior of the alloy becomes more prominent during high-temperature stretching. Gopinath et al. [50] observed that at elevated temperatures, the interaction between solute atoms and movable dislocations is more likely to lead to serrated flow. During plastic deformation, the motion of dislocations is hindered by various obstacles, including solute atoms and precipitated phases. As high-temperature stretching progresses, the diffusion rate of solute atoms accelerates, and the dislocation density caused by deformation also increases sharply. At this time, it is easier to accumulate a sufficient number of solute atoms around the dislocation to pin it down [48]. The obstruction of dislocation motion causes an increase in stress. When the dislocation is detached from the pinning, the stress decreases and the serrated flow phenomenon occurs.

**Figure 10:**
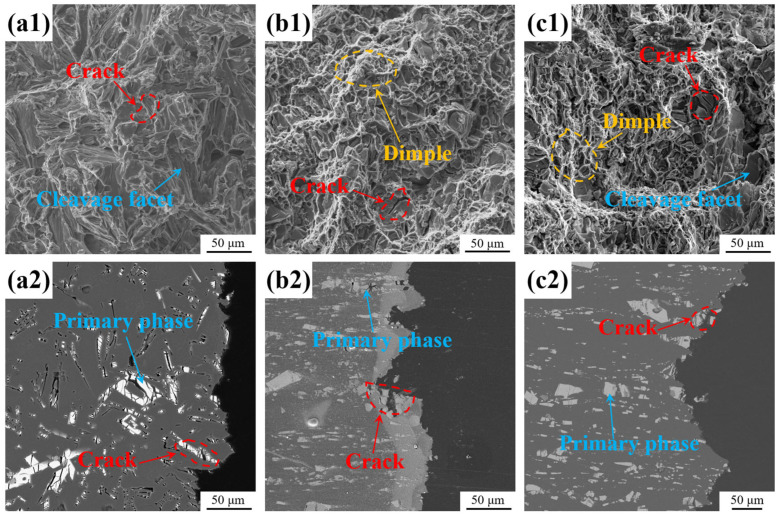
SE-SEM images of the fracture surfaces of the (**a1**) as-cast, (**b1**) as-extruded, and (**c1**) heat-treated alloys; BSE-SEM images of the longitudinal-sectional areas beneath the tensile fracture surfaces: (**a2**) as-cast, (**b2**) as-extruded, and (**c2**) heat-treated alloys.

The tensile fracture morphologies of all the alloys were analyzed using secondary electron (SE)-SEM (Figure 10(a1,b1,c1)). Instead of dimples, plenty of elongated cleavage planes and cracks were present in the fracture morphology of the as-cast alloy (Figure 10(a1)), suggesting that the fracture mode was characteristic of brittle fracture. The cleavage planes and cracks primarily resulted from the fracture of the brittle, coarse intermetallic compound. However, the fracture surface of the as-extruded alloy (Figure 10(b1)) displayed numerous dimples of various sizes, showing the typical characteristics of ductile fractures. This is in accordance with the favorable elongation (~11%) of the alloy. The substantial disparity in the dimensions of the intermetallic compound following extrusion gives rise to the formation of tough nests of different sizes. The larger dimples are associated with the fracture of micron-sized intermetallic compounds, whereas the smaller dimples correspond to the fracture of sub-micron-sized intermetallic compounds. In addition, the fracture morphology of the heat-treated alloy displayed cracks and dimples of varying dimensions, indicating that the fracture mode of the alloy remained ductile.

In order to further investigate the fracture behavior of the alloys, the longitudinal-sectional areas beneath the fracture surfaces were characterized (Figure 10(a2,b2,c2)). In the as-cast alloy, numerous cracks were observed in the intermetallic compound near the fracture surface (Figure 10(c2)). There was strong interfacial bonding between the α-Al and Al_11_Ce_3_ intermetallic compound. With stress loading, cracks propagate to interconnections, leading to the fracture of the alloy. In the as-extruded alloy, a high density of microcracks was only present in the micron-sized intermetallic compound, with no such cracks being observed in the sub-micron-sized intermetallic compound. No intermetallic compound was debonded with the matrix. These cracks are likely to have originated from the microcracks that were present in the sample after the hot extrusion process (Figure 2f). When subjected to tensile stress, microcracks in the micron-sized phase widen and tear the matrix, subsequently interconnecting to cause the fracture of the alloy. Microcracks were also observed in the micron-sized phases of the heat-treated alloy. Nevertheless, the presence of a considerable number of nano-scale strengthening phases in the heat-treated alloy results in an enhanced matrix. Under stress loading, the expansion of microcracks is impeded by the matrix. As the stress continues to increase, a significant number of cracks expand simultaneously, leading to a decrease in the elongation of the alloy.

## 4. Discussion

### 4.1. Intermetallic and Microstructure Evolutions

The as-cast Al-10Ce-3Mg-5Zn alloy is composed of α-Al and coarse bulk intermetallic compound, as well as fine intermetallic compound clusters. During the process of hot extrusion, the fine intermetallic compound is extruded into small particles, while the coarse intermetallic compound is not completely broken down, and high-density microcracks are formed on their surfaces. The size distribution of the intermetallic compounds exhibits a bimodal shape. Moreover, the bimodal particles show a lamellar distribution as the Al matrix flows. Compared with the equiaxed grain size of the as-cast alloy, the extruded alloy’s grain size is refined due to recrystallization. During the extrusion process, regions with high dislocation density and large orientation gradients form near the intermetallic compound. The deformation zones provide the necessary driving force for recrystallization. In addition, as the strain increases, dislocations become progressively tangled and eventually evolve into grain boundaries, which promotes the formation of finer, equiaxed grains in the as-extruded alloy (Figure 4b). The fine and coarse grains are situated in close proximity to the large-scale and small-scale phases, respectively, and the fine and coarse grain layers are alternately distributed. A possible mechanism behind this is that the uneven deformation near the bimodal particles leads to the difference in nucleation energy [41]. In contrast to fine intermetallic compounds, severe deformation is more pronounced near coarse intermetallic, providing both higher nucleation energy and additional sites for recrystallization. The final recrystallized grain size is determined by the balance between nucleation and grain growth. When the rate of nucleation surpasses that of grain growth, a smaller grain size is observed. The probability of nucleation of recrystallization around the coarse phases is greater than it is around the fine phases. Furthermore, the large-scale phases have a more pronounced pinning effect on the migration of grain boundaries. Consequently, during the water-cooling process, the growth of recrystallized grains is greatly hindered, resulting in the large-scale phase being enclosed by fine grains. In contrast, the small-scale phase does not exhibit similar behavior, resulting in the grains being larger near the phase.

It is worth noting that twins are clearly observed in the phases of the alloy. It was shown that the phase transition temperature of pure Al_11_Ce_3_ is 1006 °C, and the high-temperature phase β-Al_11_Ce_3_ (commonly referred to as Al_4_Ce) is transformed into the low-temperature phase α-Al_11_Ce_3_ during the cooling process. The α-Al_11_Ce_3_ phase is orthorhombic (Immm), while the β-Al_11_Ce_3_/Al_4_Ce phase is tetragonal (I_4_/mmm, D13). Qi et al. [51] found twins in the arc-melted Al11Ce3 phase, which were presumed to be formed after cooling during the allotropic β-α phase transition. Usually, the eutectic Al-Ce alloy will directly form α-Al_11_Ce_3_ during solidification. However, studies have shown that the Al_11_Ce_3_ phase prepared by means of additive manufacturing was transformed into the Al_4_Ce phase after heating at 400 °C for 1 h [52]. In this study, the α-Al_11_Ce_3_ phase was transformed into the β-Al_11_Ce_3_ phase during the hot extrusion of the Al-10Ce-3Mg-5Zn alloy at 450 °C. The formation of twins is induced by the β-α phase transformation that takes place during the alloy’s cooling process.

After heat treatment, numerous nano-scale strengthening T phases were precipitated in the as-extruded alloy. The EPMA maps of the alloy (Figure 3c) clearly show that the Mg element is dissolved within the α-Al matrix. Most of the Zn element is enriched in the Al_11_Ce_3_ phase, leading to the formation of the Al_2_CeZn_2_ phase, while the remaining Zn is dissolved in the α-Al matrix. The alloy obtains double-super-saturated vacancies and a solid solution after quenching. During the aging process, the solute atoms Mg and Zn cluster rapidly to form the GP (Guinier–Preston) zone. Increasing the aging time, the GP zone is transformed into the transition T′ phase. Finally, a stable phase, T-Mg_32_(Al Zn)_49_, is formed to enhance the strength of the α-Al matrix [53,54].

### 4.2. Strengthening Mechanism

The strength of the as-cast Al-10Ce-3Mg-5Zn alloy was significantly enhanced following the hot extrusion and subsequent heat treatment. In general, the strengthening mechanisms of alloys containing particle strengthening phases can be classified into five types: (I) fine grain strengthening; (II) dislocation strengthening; (III) solid solution strengthening; (IV) Orowan strengthening; and (V) load transfer strengthening [35]. According to previous studies, load transfer strengthening is an important strengthening mechanism for alloys containing particle strengthening phases [55,56]. However, in this study, the coarse intermetallic compound of the alloy was found to be surrounded by high-density microcracks after the hot extrusion. Zhang et al. [41] revealed that the load transferred by the α-Al matrix promoted the propagation of microcracks within the intermetallic phases, leading to the premature yield of these compounds. Therefore, the effect of load transfer strengthening was not considered in the present study.

The EBSD results indicate that the as-extruded alloy exhibits notable grain refinement as a consequence of recrystallization (Figure 4). There is no obvious change in the grain size of the alloy following heat treatment. The Hall–Petch equation presented below can be employed to assess the impact of fine grain strengthening [57,58,59]:

(1) $$\sigma_{g}=\sigma_{0}+Kd^{-1/2}$$

where *σ*_0_ is the lattice friction force of aluminum, and *K* and *d* are the Hall–Petch coefficient and average grain size, respectively [57]. The refinement of grains results in an increased density of grain boundaries within the alloy, which effectively obstructs dislocation motion and thereby enhances the alloy’s strength.

It has been demonstrated that dislocations generated during hot extrusion can enhance the strength of an alloy. The Taylor equation provides a means to explain the correlation between the increase in strength and dislocation density (*ρ*) [60]:

(2) $$\sigma_{d}=M\alpha bG\sqrt{\rho }$$

where *α* is the dislocation strengthening, *G* is the shear modulus, *b* is the length of the Burgers vector in Al, and *M* is the Taylor factor of Al [61]. The dislocation density (*ρ*) can be determined from the EBSD data, as shown in Figure 5. The dislocation densities in the as-extruded and heat-treated alloys are approximately equivalent, resulting in a comparable dislocation strengthening effect for the two alloys.

The alloy contains a significant amount of solid solution elements, with Zn and Mg being the primary constituents, while the combined content of other elements remains below 0.5 wt.%. As a result, the solid solution strengthening effect of the alloy must be considered. The strengthening contribution from the Zn and Mg elements can be calculated using the following formula [62]:

(3) $$\sigma_{s}=\sum k_{j}c_{j}^{m}$$

where *k_j_* is the reinforcement constant of the solute element *j*, *c_j_* is the concentration of the solute element *j*, and *m* is taken as 1. However, during the solidification process of the alloy, some of the Zn will accumulate in the intermediate Al_11_Ce_3_ phase to form the Al_2_CeZn_2_ phase. After heat treatment, a considerable quantity of Zn and Mg will be converted into the secondary precipitate T phase, leading to a decrease in the solute element concentration within the matrix. As a result, the value of the actual solid solution strengthening effect is lower than the theoretical value.

The Orowan strengthening effect is contingent upon the number and size of precipitates [63]. Reducing the precipitate size or increasing their quantity can lead to an improvement in the strength of an alloy. The calculation formula for Orowan strengthening is shown below [64]:

(4) $$\sigma_{o}=M\frac{0.4Gb}{\pi \sqrt{1-v}}\frac{\ln(2\bar{r}/b)}{\lambda_{p}}$$

where *b* is the Burgers vector, *M* is the average orientation factor, *G* is the shear modulus of Al, and *v* is Poisson’s ratio. *λ_p_* represents the average precipitation spacing. $\bar{r}$ is the average diameter of the statistical measurement, calculated by the following formula [65]:

(5) $$\bar{r}=\sqrt{2/3}r$$

where *r* is the average radius of the precipitates. Following heat treatment, a significant quantity of T phases are precipitated in the alloy, significantly improving its strength.

In conclusion, the reduction in grain size and the increase in dislocation density observed in the as-cast alloys following hot extrusion result in an improvement in both fine grain strengthening and dislocation strengthening effects. The composition of solute atoms in the as-extruded alloy remains largely unchanged, resulting in a consistent contribution from solid solution strengthening. In particular, a substantial quantity of secondary T phase precipitates forms in the alloy after heat treatment. The precipitation of some solid solution atoms in the heat-treated alloy leads to a reduction in the solid solution strengthening effect. However, precipitation strengthening remains the most effective method for enhancing the strength of an alloy. The high-density nano-precipitates will impede the dislocation movement, resulting in a pronounced increase in the Orowan strengthening effect. As a result, the strength of the heat-treated alloy is substantially enhanced by the combined action of the four strengthening mechanisms.

### 4.3. Ductility

The elongation of the as-cast alloy increases significantly after hot extrusion. Meanwhile, following heat treatment, the plasticity of the as-extruded alloy decreases, although it remains higher than that of the as-cast alloy. These changes in ductility can be attributed to alterations in grain size and structural changes in both the α-Al matrix and Al_11_Ce_3_ phase.

Firstly, the grain sizes of both the as-extruded and heat-treated alloys are significantly finer compared to the as-cast alloy. Grain refinement effectively enhances ductility, while the high-density grain boundaries help to hinder crack propagation, thus improving damage tolerance. Furthermore, the alternating layered structure of the coarse and fine grain layers in the alloy contributes to an improvement in plasticity [66]. It has been demonstrated that coarse grains exhibit a greater dislocation storage capacity and superior plasticity in comparison to fine grains [67]. When the crack encounters the coarse grain layer, the layer effectively inhibits its propagation by blocking the crack’s growth. In the present study, the grains of the alloys exhibit finer sizes in the coarse intermetallic compound region and larger sizes in the fine intermetallic compound region. Concurrently, the surface of the coarse intermetallic compound following hot extrusion is characterized by a high density of microcracks, leading to the initial crack formation in the fine grain region. When subjected to stress, these cracks propagate from the fine grain zone toward the coarse grain zone, where it will be delayed. This process enhances the plasticity of the alloy.

**Figure 11:**
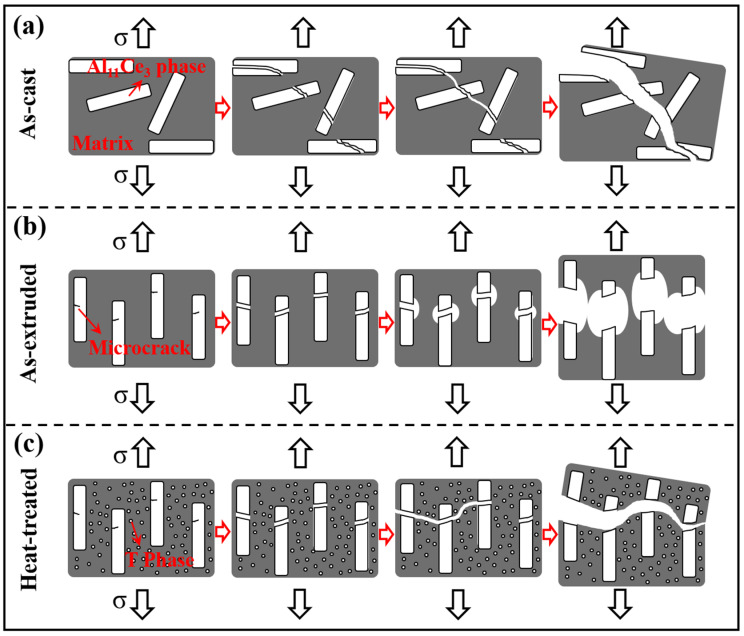
Schematic diagram of the tensile deformation process of the (**a**) as-cast, (**b**) as-extruded, and (**c**) heat-treated alloys.

Secondly, the fracture behavior of the alloy is sensitive to the microstructure [68]. When the angle between the lamellar phase and the applied load direction differs, the crack exhibits different propagation paths, resulting in disparate fracture modes. Cracks are easily formed in the bulk Al_11_Ce_3_ phase under stress loading [41]. When the tensile axis is aligned perpendicular to the lamellar Al_11_Ce_3_ phase, the cracks expand rapidly along the lamellar plane, resulting in the cleavage fracture of the alloy. When the tensile axis is parallel to the lamellar Al_11_Ce_3_ phase, the alloy exhibits plastic fracture with dimples. In the as-cast Al-10Ce-3Mg-5Zn alloy, the intermetallic compound is distributed in a random manner. When subjected to stress, cracks in intermetallic compounds that are perpendicular to the load direction will expand rapidly (Figure 11a). Due to the high interfacial bonding strength between the intermetallic compound and the α-Al matrix and the softness of the α-Al matrix, the cracks expand and tear the matrix, resulting in brittle fracture of the alloy (Figure 10a). Following the hot extrusion process, a portion of the large-scale phases within the alloy are refined into small-scale phases, while the large-scale phases with microcracks are oriented parallel to the load direction (Figure 2b). During tensile deformation, the pre-existing microcracks in the large-scale phase expand and become wider (Figure 11b). The hardening process facilitates the plastic deformation of the α-Al matrix near the crack tip, thereby impeding the propagation of the crack. Concurrently, microcrack propagation and twin deformation will absorb energy and alleviate the stress generated by the mismatch in deformation between the hard particles and the soft matrix. The plasticity of the alloy is enhanced, and the alloy exhibits plastic fracture with dimples (Figure 10b). After heat treatment, the pre-existing microcracks will also expand and widen under stress loading. The difference is that due to the formation of numerous nano-strengthened T phases in the matrix after heat treatment, the plastic deformation ability of the matrix decreases sharply with the increase in strength. When the cracks extend to the aluminum matrix, it is difficult for the matrix to undergo plastic deformation to release energy (Figure 11c). The tensile stress further increases, and a large number of cracks expand and tear the matrix, ultimately leading to the fracture of the alloy (Figure 10c). Although the plasticity of the heat-treated alloy decreases, its fracture mode remains predominantly plastic.

## 5. Conclusions

In this study, a novel Al-10Ce-3Mg-5Zn heat-resistant alloy with high strength and high plasticity was prepared by the hot extrusion and heat treatment of the as-cast alloy. Then, this paper expounds on the relationship between microstructure and mechanical properties and offers suggestions for enhancing the properties of the Al-Ce alloys. The conclusions are as follows:

(1) The as-extruded alloy exhibits a bimodal intermetallic compound, and there are high-density microcracks and twins in the coarse intermetallic compound. Furthermore, the bimodal intermetallic compound structure gives rise to the bimodal distribution of α-Al grains. In addition to the formation of a substantial number of strengthened nano-scale T phases, there is no discernible alteration in the other microstructures of the heat-treated alloy.
(2) Due to the combined effect of grain boundary strengthening and dislocation strengthening, the as-extruded alloy has higher UTS, which is 51% higher than that of the as-cast alloy at 210 MPa, reaching 318 MPa. After heat treatment, the strength of the alloy is further improved to 367 MPa, primarily due to the enhanced Orowan strengthening effect resulting from the precipitation of the nano-strengthened T phase.
(3) The as-extruded alloy displays notable plasticity, which is primarily attributable to the bimodal structure of the alloy, the orientation relationship between the intermetallic phase and the load, the pre-existing microcrack propagation, and twin deformation. The plasticity of the heat-treated alloy is diminished, primarily due to the T phase strengthening of the matrix and the simultaneous reduction in its plastic deformation ability.
(4) The heat-treated alloy exhibits a good strength retention rate at elevated temperatures. The UTS retention rate of the heat-treated alloy at room temperature was calculated to be 64% at 200 °C, 29% at 260 °C, and 21% at 300 °C. Moreover, the room- and elevated-temperature stress–strain curves of the studied alloys were serrated. The primary factors contributing to the serrated flow behavior are twinning deformation, the interaction between the obstacles (such as solute atoms and precipitated phases), and movable dislocations.

## Figures and Tables

**Table 1:** EPMA results for the points illustrated in Figure 3 (at.%).

Point	Al	Ce	Mg	Zn	Zr	Y	Phase
1	69.64	20.07	0.15	9.79	0.01	0.34	AlCeZn
2	70.68	20.93	0.05	8.06	0.01	0.27	AlCeZn
3	84.42	4.53	5.37	4.31	1.36	0.01	AlCeMgZn
4	83.30	4.31	6.64	4.34	1.37	0.04	AlCeMgZn

## Data Availability

The original contributions presented in the study are included in the article, further inquiries can be directed to the corresponding author.
